# A New Remedy, Called Digestine

**Published:** 1877-03

**Authors:** A. F. Shelly

**Affiliations:** Philadelphia


A New Remedy, Called Digestine.—*By A. F. Shelly, M.D., of Philadelphia.*—This is obtained from the gizzard of the domestic fowl (chicken), and is a specific for vomiting in pregnancy. I have used this remedy for twenty-five years, and it has never failed. It is also the most powerful and reliable remedy for the cure of indigestion (dyspepsia), and sick stomach caused from debility of that organ. It is useful in all cases where the pepsines and pancreatines are used, but with much more certainty of its good results, for it puts all these preparations, in my experience, in the back-ground.

In complicated affections of the stomach, such as inflammation, gastralgia, pyrosis, etc., it may be combined with subnitrate of bismuth and opiates; and in diarrhœa and cholera infantum, with astringents, both vegetable and mineral. I have given the article to several prominent physicians, who have used it with the happiest results, among whom I may mention Professor E. Wallace, of the Jefferson Medical College; he gives me the result of seventeen cases as follows:

In vomiting of pregnancy, out of nine cases he cured six, and palliated two, and in one case the remedy was not taken according to direction, and therefore had no effect.

He used it in seven cases of sick stomach caused by chronic inflammation of the uterus; cured five, and two remained doubtful. He also used it in a case of very obstinate sick stomach, caused by an irreducible hernia, and says this was the only remedy that gave any relief.

We, who have some experience, all know that vomiting of pregnancy is a sore affliction, and in some cases almost unendurable, nay, indeed, putting life in jeopardy; but in digestine we have a remedy which will prove to be a great blessing to mothers, who, as yet, think vomiting must be endured as a natural conseqence.

If I am able, by this publication, to induce the medical fraternity to make use of the remedy, I am positive that a great boon will be conferred upon a class of sufferers who claim our sympathy.

The dose is from five to ten grains, hardly ever more than five, except in obstinate cases. For children, from one to five grains. My mode of administering it is in a spoonful of water or tea, or it may be strewn on a piece of bread and covered over with a little butter; it is, however, nearly tasteless. In dyspepsia and in vomiting of pregnancy, I direct it to be taken half an hour or so before each meal. In other affections of the stomach and bowels, every two to four hours. I

give it uncombined, except in complicated cases, as heretofore mentioned.

The methods by which this principle can be obtained from the viscus are various. When I commenced to employ it, I used it in rather a crude state, by pulverizing the lining membrane of the gizzard; but it requires too much care and precision in the drying and cleansing operation, in order not to destroy its virtues. There is also great inconvenience in obtaining the viscus during the heat of summer and extreme cold of winter, as temperature is one of the main things to be observed, in order to preserve its efficacy, purity and sweetness. Later, finding this mode of preparation unsatisfactory, and inconvenient for the above reasons, I consulted with Wm. R. Warner & Co., 1228 Market street, Philadelphia, who have prepared a form, designated digestine; its purity, and also its good effects, I can vouch for.—*Med. and Surg. Reporter.*

